# Chromosome-level genome assembly of ridgetail white shrimp *Exopalaemon carinicauda*

**DOI:** 10.1038/s41597-024-03423-9

**Published:** 2024-06-04

**Authors:** Jiajia Wang, Jianjian Lv, Miao Shi, Qianqian Ge, Qiong Wang, Yuying He, Jian Li, Jitao Li

**Affiliations:** 1 State Key Laboratory of Mariculture Biobreeding and Sustainable Goods, Yellow Sea Fisheries Research Institute, Chinese Academy of Fishery Sciences, Qingdao, Shandong 266071 China; 2 Laboratory for Marine Fisheries Science and Food Production Processes, Qingdao Marine Science and Technology Center, Qingdao, Shandong 266237 China; 3 Berry Genomics Co., Ltd., Beijing, China

**Keywords:** Genome, Genome informatics, Functional genomics

## Abstract

*Exopalaemon carinicauda*, a eurythermal and euryhaline shrimp, contributes one third of the total biomass production of polyculture ponds in eastern China and is considered as a potential ideal experimental animal for research on crustaceans. We conducted a high-quality chromosome-level genome assembly of *E. carinicauda* combining PacBio HiFi and Hi-C sequencing data. The total assembly size was 5.86 Gb, with a contig N50 of 235.52 kb and a scaffold N50 of 138.24 Mb. Approximately 95.29% of the assembled sequences were anchored onto 45 pseudochromosomes. BUSCO analysis revealed that 92.89% of 1,013 single-copy genes were highly conserved orthologs. A total of 44, 288 protein-coding genes were predicted, of which 70.53% were functionally annotated. Given its high heterozygosity (2.62%) and large proportion of repeat sequences (71.49%), it is one of the most complex genome assemblies. This chromosome-scale genome will be a valuable resource for future molecular breeding and functional genomics research on *E. carinicauda*.

## Background & Summary

The family Palaemonidae, including more than 1400 species in 181 genera, represents the largest family of the order Decapoda^1^. Animals from this family are found in marine and freshwater environments in tropical to temperate regions worldwide. It includes several shrimps with high economic value, such as *Macrobrachium rosenbergii*, *Macrobrachium nipponense* and *Exopalaemon carinicauda*. The ridgetail white shrimp *E. carinicauda* is a eurythermal and euryhaline shrimp distributed over a wide geographical area throughout tropical, subtropical, and temperate coastal waters^2,3^. It can survive in a multitude of environmental extremes, has a broad salinity tolerance of 2–44 and can survive in freshwater after domestication^4^. It is also capable of inhabiting temperatures as low as −3 °C and as high as 39 °C^5,6^. As one of the most commercially valuable pond-raised species of shrimp, *E. carinicauda* contributes to one third of the total production of polyculture ponds in eastern China^7^.

**Fig. 1:**
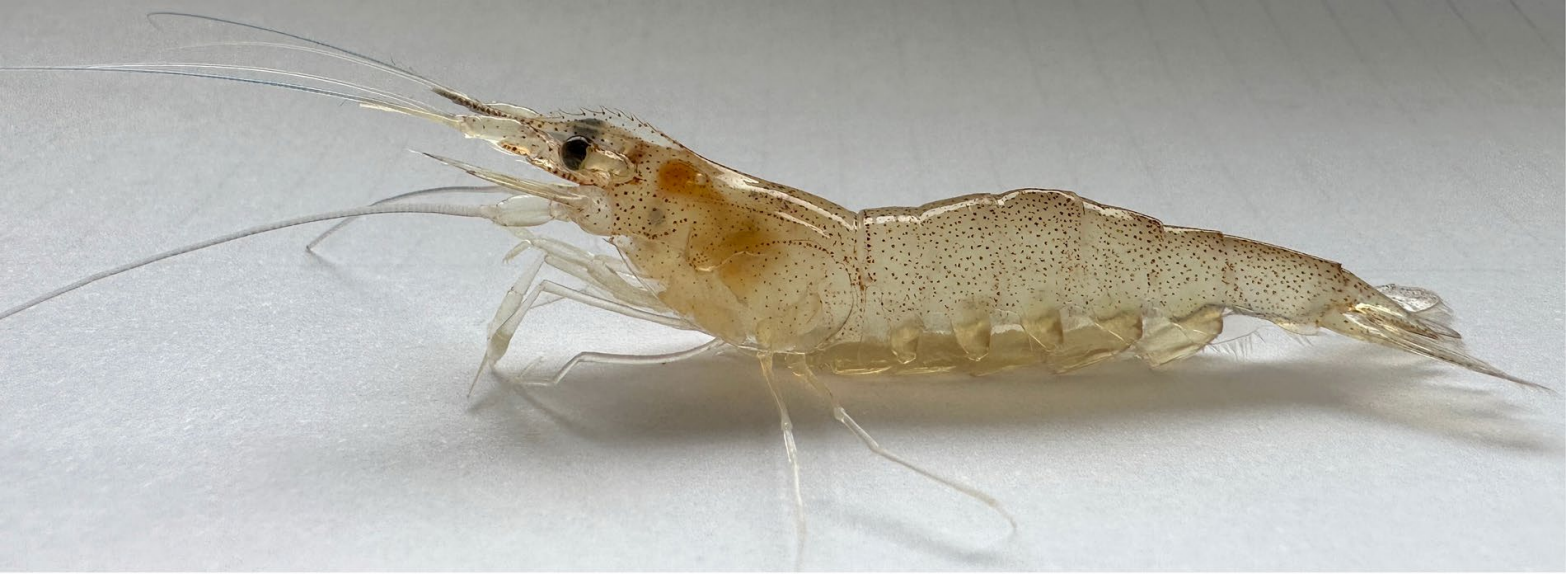
A lateral full-body view of the sequenced *E. carinicauda*.

In addition to its important economic value in aquaculture, it is considered a potential ideal experimental animal for research on crustaceans for its moderate size, transparent body (Fig. 1), short reproductive cycle, large eggs (diameters ranging 0.57–1.08 mm) and ease of culturing and breeding in captive conditions^8^. Currently, CRISPR/Cas9-mediated genome editing technology has been successfully used in *E. carinicauda*, which is the first time that gene editing has been realized in a decapod crustacean^9,10^. However, the absence of genomic data limits the further application of gene editing in studying the molecular biology, cytobiology and genetics of crustaceans. Therefore, a high-quality reference genome is essential for understanding the molecular biology, genetics, breeding, ecology and adaptation of *E. carinicauda*.

A fragmented draft genome of *E. carinicauda* has been assembled using Illumina short reads containing 13,897,062 scaffolds (contig N50, 263 bp)^11^. Genome survey analysis indicated that *E. carinicauda* has a relatively large genome size of 5.73 Gb, which is at least twice as large as that of many decapod shrimps^12–14^. In this study, an improved chromosome-level genome of *E. carinicauda* was assembled using the PacBio sequencing platform, Illumina paired-end sequencing, and high-throughput chromatin conformation capture (Hi-C) technology. Our previous studies suggested that the *E. carinicauda* karyotype is 2n = 90^15^, similar to that of other *Exopalaemon* species^16^. The final genome size was 5.86 Gb with a contig N50 length of 235.52 kb and a scaffold N50 length of 138.24 Mb. A total of 44,288 protein-coding genes were predicted in the genome of *E. carinicauda*. This chromosome-level genome assembly of *E. carinicauda* provides a valuable genomic resource for further genetic improvement and understanding of the functional genes and molecular mechanisms of *E. carinicauda*.

## Methods

### Animal materials and genome sequencing

A female shrimp was collected from Rizhao Haichen Aquatic Co., Ltd. The muscle tissue was collected for DNA extraction and library construction. Total genomic DNA was extracted using a cetyltrimethylammonium bromide method. For the genome survey, a 350 bp paired-end library was constructed according to the manufacturer’s instructions (Illumina, San Diego, CA, USA) and sequenced on an Illumina NovaSeq 6000 platform. A total of 276.18 Gb of raw data were obtained, which covered approximately 54 × of the estimated genome (Table 1).

**Table 1:** Genome assembly statistics of *E. carinicauda*.

Items	Values
Estimated genome size (Mb)	5,126.74
Heterozygosity	2.62%
Repeat rate	84.74%
PacBio assembly	
Assembled genome size (Mb)	5,857.36
Contig N50 (bp)	235,277
Contig N90 (bp)	48,014
Number of contigs	47,421
Max length (bp)	3,038,493
GC content	34.79%
Hi-C assembly	
Assembled genome size (Mb)	5,581.49
N50 (bp)	138,242,434
Scaffold number	4,022
Max length (bp)	338,475,513
Pseudochromosome number (2n)	90
Unplaced scaffold (Mb)	275.87
Place rate	95.29%

For PacBio sequencing, a 15 kb library was constructed using the SMRTbell Express Template Prep Kit 2.0 (Pacific Biosciences, Menlo Park, CA, USA) and sequenced with circular consensus sequencing mode using a single 8 M SMRT Cell on the PacBio Sequel II platform (Pacific Biosciences). After filtering out the low-quality reads and sequence adapters, 3636.91 Gb subreads of PacBio Data were obtained, representing approximately 708 × sequence coverage based on the estimated genome size (Table 1). Finally, 203.27 Gb of CCS reads were generated using SMRTLink 9.0 which covered approximately 40 × of the estimated genome.

For the construction of the Hi-C library, DNA was fixed with 4% formaldehyde solution and digested with the 4-cutter restriction enzyme MboI. The digested fragments were labeled with biotin-14-dCTP, then the cross-linked fragments were subjected to blunt-end ligation. The library was sequenced on the Illumina NovaSeq 6000 platform, and approximately 552.65 Gb of Hi-C clean reads were generated, covering approximately 108 × of the estimated genome (Table 1).

### Genome survey

**Fig. 2:**
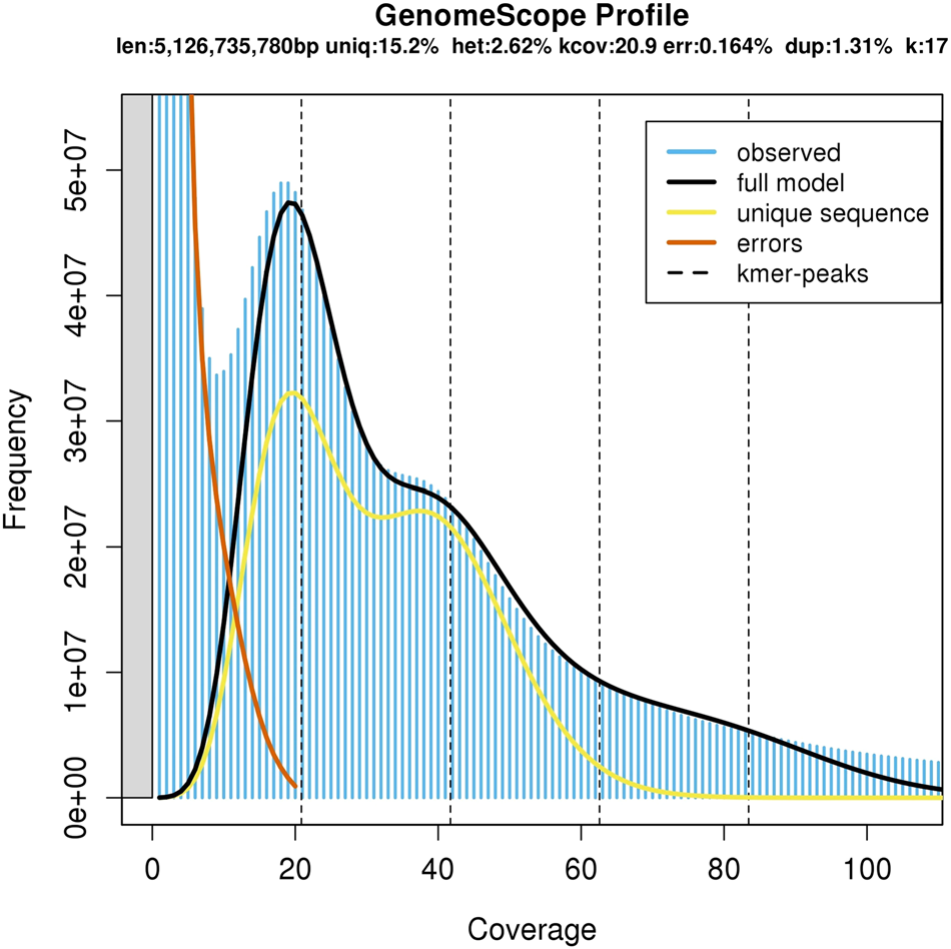
The 17-mer analysis of the genome.

The genome size and heterozygosity were estimated using the k-mer method before genome assembly^17^. The k-mer distribution was calculated from Illumina short reads using Jellyfish based on k-mer (k = 17)^18^. The heterozygosity ratio was estimated by the online tool of GenomeScope^19^ (https://github.com/schatzlab/genomescope). Finally, the estimated genome size of *E. carinicauda* was predicted to be approximately 5.12 Gb, with 84.74% repetitive sequences, and the genome heterozygosity was 2.62% using a 17-mer analysis (Fig. 2), suggesting a complex genome of *E. carinicauda*.

### Chromosome-level genome assembly

The initial genome was assembled with HiFi reads using the Peregrine (v0.1.6.1) (https://github.com/cschin/peregrine). A modified “best overlap graph” strategy was used to get the contig assembly based on the overlap graph. Contig overlaps were removed from the assembled contig sequences using Purge_dups (https://github.com/dfguan/purge_dups). *De novo* assembly of PacBio sequences yielded a preliminary assembly of 5.86 Gb, containing 47,421 contigs with a contig N50 length of 235.28 kb, a maximum length of 3,038,493 bp and a GC content of 34.79% (Table 1).

**Fig. 3:**
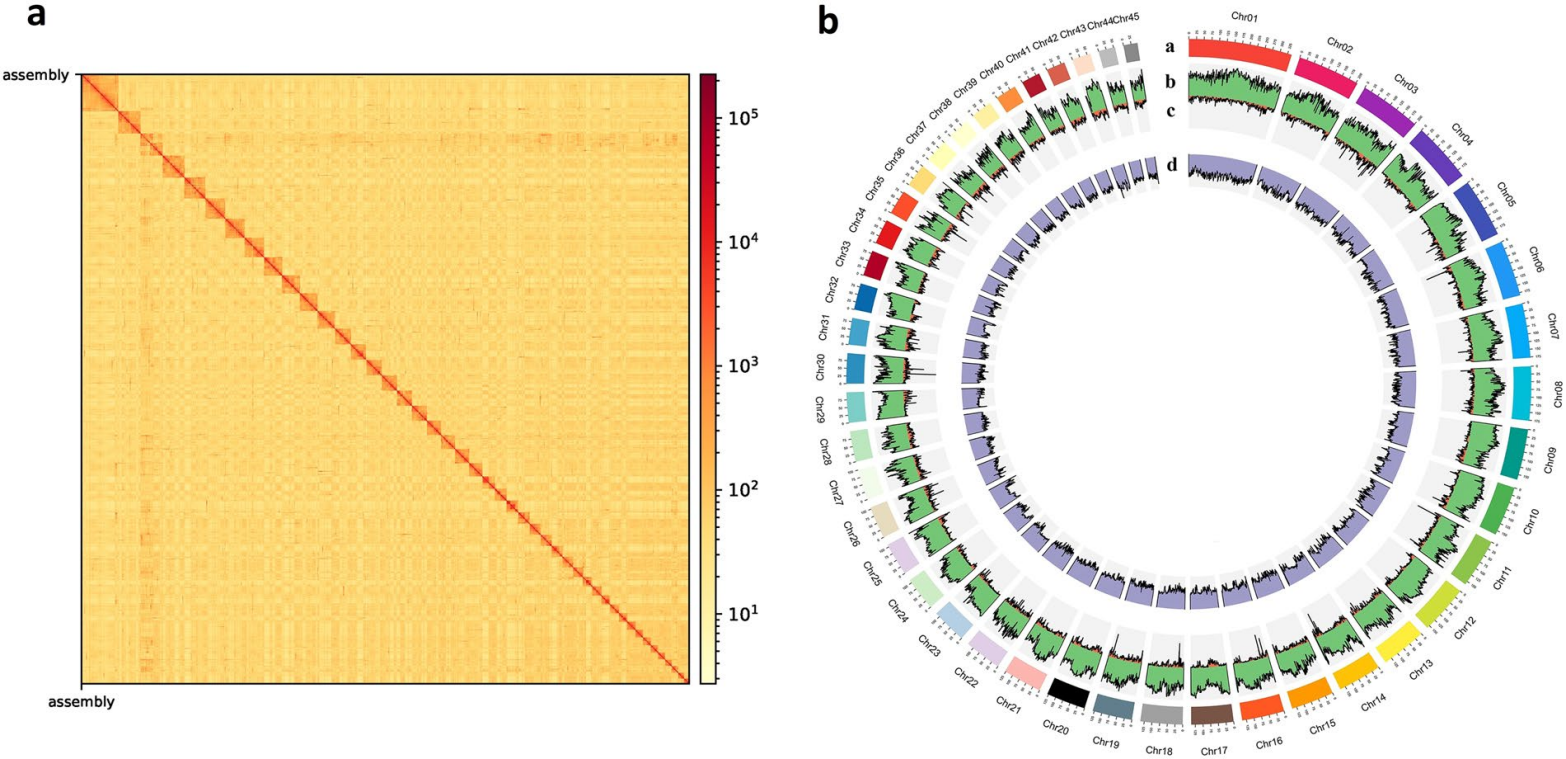
Genome assembly of *E. carinicauda*. (**a**) Hi-C assembly of chromosome interactive heatmap. A deeper colour represents stronger interaction between contigs. (**b**) Characterization of assembled genome. a, Physical map of *E. carinicauda* pseudochromosomes (Mb scale), different colour represents different chromosome. b, proportional distribution of repeated sequences in 1 Mb window. c, gene density represented by number of genes in 1 Mb window. d, GC content represented by percentage of G/C bases in 1 Mb window.

Chromosome-level assembly of *E. carinicauda* was conducted using Hi-C technology. Juicer (v1.6.2)^20^ and 3D-DNA (v180922)^21^ software were implemented to obtain the chromosome-level whole genome assembly. The filtered Hi-C reads were aligned to the initial draft genome using Juicer (v1.6.2). Only uniquely mapped and valid paired-end reads were used for the assembly using 3D-DNA. Juicebox (v1.9.8) was used to manually order the scaffolds to generate more precise chromosome-level genome of *E. carinicauda* according to the chromosomal interaction heatmap^22^. Contact maps were visualized using HiCExplorer (v3.3)^23^. The number of chromosomes was 90, which was determined based on karyological observations of *E. carinicauda* chromosomes in our previous study^15^. The contigs were ultimately clustered into 45 pseudochromosomes for *E. carinicauda*, with a scaffold N50 length of 138.24 Mb. The total length of the 45 pseudochromosomes was 5.58 Gb (covered 95.29%) (Fig. 3a,b), of which the length ranged from 46.25 Mb to 338.48 Mb. The length of the un-placed scaffolds was 275.86 Mb (Table 2).

**Table 2:** Statistics of cluster number and length of single chromosome.

Chromosome	Chromosome Length(bp)
Chr01	338,475,513
Chr02	204,730,873
Chr03	201,960,415
Chr04	198,872,827
Chr05	193,192,193
Chr06	183,704,020
Chr07	177,419,726
Chr08	177,097,468
Chr09	166,825,026
Chr10	163,202,111
Chr11	160,936,739
Chr12	159,029,458
Chr13	148,231,718
Chr14	143,083,519
Chr15	140,275,458
Chr16	138,927,385
Chr17	138,242,434
Chr18	137,840,206
Chr19	130,123,217
Chr20	129,145,531
Chr21	127,801,978
Chr22	121,442,373
Chr23	114,382,935
Chr24	109,748,301
Chr25	107,571,620
Chr26	105,404,600
Chr27	100,157,323
Chr28	99,937,579
Chr29	99,510,691
Chr30	99,154,881
Chr31	87,323,507
Chr32	86,904,133
Chr33	84,323,655
Chr34	81,141,169
Chr35	80,118,929
Chr36	76,002,002
Chr37	75,759,974
Chr38	70,568,037
Chr39	69,120,708
Chr40	68,560,120
Chr41	61,475,706
Chr42	61,441,371
Chr43	61,143,013
Chr44	54,930,122
Chr45	46,253,857

**Fig. 4:**
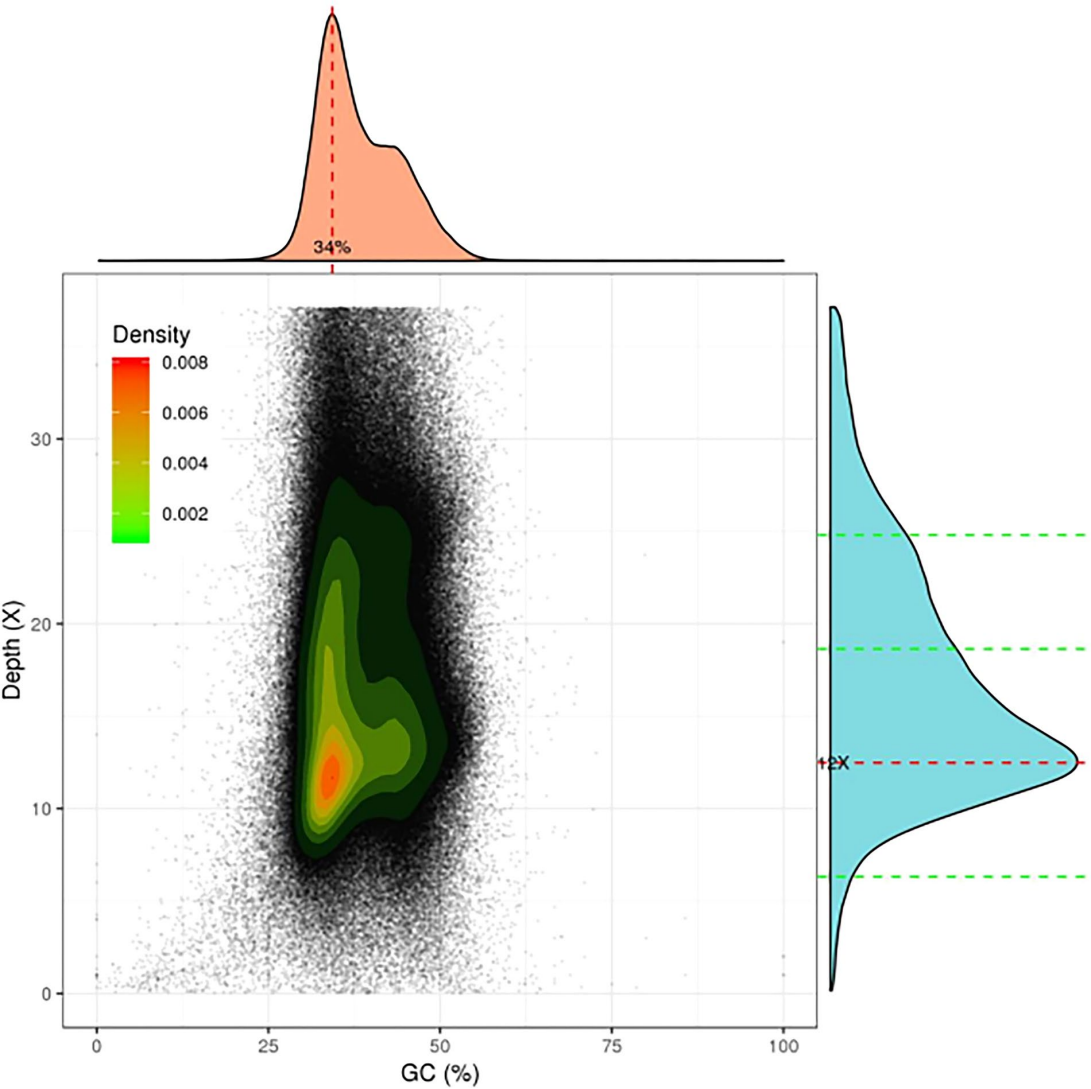
GC content and depth distribution. The horizontal axis represents the percentage of GC content, and the vertical axis represents the average sequencing depth.

The quality of the final chromosome-level genome assembly was assessed using the following three methods. First, we aligned the Illumina DNA short reads obtained from our previous study to the assembled genome and found that approximately 99.00% of the DNA short reads could be mapped to our assembly using BWA (v0.7.15)^24^. Second, read depth and GC content with 10 kb windows were used to evaluate the assembly results and determine whether there was a significant GC bias or sample contamination, showing that the assembled genome was clean without contamination (Fig. 4). Finally, genome assembly and completeness were further evaluated using conserved genes in benchmarking universal single-copy orthologs (BUSCO, v5.2.2) with the arthropoda_odb10 database^25^. The results showed that 92.89% of the 1013 single-copy genes were highly conserved orthologs (88.75% complete, 4.15% fragmented, and 7.11% missing) (Table 3).

**Table 3:** Universal single copy ortholog (BUSCO) assessment of *E. carinicauda*.

	Number	Percent
BUSCOs	941	92.89%
Complete BUSCOs	899	88.75%
Fragmented BUSCOs	42	4.15%
Missing BUSCOs	72	7.11%
Total BUSCO groups searched	1013	100%

Compared to the published genome of *E. carinicauda*^11^, our assembled genome is of significantly improved quality and integrity. The contig N50 increased from 263 bp to 235,277 bp, with an increase of nearly 900-fold, and scaffold N50 increased from 816 bp to 138,242,434 bp. Meanwhile, the assembled complete orthologue proportion enhanced from 43.44% to 88.75% according to the BUSCO assessment.

### Repetitive and non-coding gene prediction

To detect repeat elements in *E. carinicauda* genome, *de novo* and homology-based strategies were combined using multiple methods. Mini-inverted repeat transposable elements (MITEs) were identified using MITE-Hunter (v1.0)^26^ for *de novo* annotations. Long terminal repeat sequences (LTRs) were detected using LTRharvest^27^ and LTR_Finder (v1.07)^28^, and the prediction results of these two software programs were integrated using LTR_retriever (v2.8.2)^29^. RepeatMasker (v4.1.0)^30^ was used in the homology-based alignment to search *E. carinicauda* genome sequence in the RepBase database (http://www.girinst.org/repbase). RepeatMasker was used to mask the repetitive sequences obtained by the above method, and RepeatModeler (v2.0)^31^ was used to perform the *de novo* identification of other repetitive sequences with the repeat-masked genome. Ultimately, we identified approximately 4.19 Gb of repetitive sequences, accounting for approximately 71.49% of the assembled genome, among which 9.97% were tandem repeat sequences. Among these repetitive sequences, LTRs (42.52%) accounted for the highest proportion of the assembly, followed by DNA (10.81%) and LINE (3.33%) (Table 4).

**Table 4:** Repeat components in *E. carinicauda* genome.

Repeat elements	Repeat Number	Repeat Length (bp)	Percent
LTR	9,493,987	2,492,087,017	42.52%
LINE	733,909	195,355,154	3.33%
SINE	10,552	1,254,091	0.02%
DNA	4,036,478	633,391,696	10.81%
RC/Helitron	9,285	1,630,441	0.03%
Tandem Repeat	2,211,672	584,168,817	9.97%
Unknown	1,717,222	281,865,705	4.81%
Total	18,213,105	4,189,752,921	71.49%

Five types of noncoding RNA (ncRNA) were identified in the genome of *E. carinicauda*, including microRNAs (miRNAs), transfer RNAs (tRNAs), ribosomal RNAs (rRNA), small nuclear RNAs (snRNAs) and small nucleolar RNAs (snoRNAs). The tRNA was predicted using tRNAscan-SE (v2.0)^32^. Other types of ncRNAs were detected by alignment to Rfam database^33^ using infernal (v1.1.3) software^34^. In total, 10249 non-coding RNAs (ncRNAs) were annotated, including 3,702 rRNAs, 386 miRNAs, 5,811 tRNAs, 269 snRNAs, and 81 snoRNAs (Table 5).

**Table 5:** Classification of ncRNAs in *E. carinicauda* genome.

Type	Number	Average length(bp)	Total length(bp)	Percent
rRNA	3,702	212	787,473	0.0134%
miRNA	386	109	42,080	0.0007%
tRNA	5,811	73	426,159	0.0073%
snRNA	269	144	38,829	0.0007%
snoRNA	81	191	15,538	0.0003%

### Gene prediction and annotation

We detected the protein-coding genes in the *E. carinicauda genome* assembly by a comprehensive strategy that combined *ab initio* prediction, protein-based homology searches, and RNA sequencing data predictions. For *ab initio* prediction, augustus (v3.2.2)^35^, SNAP (v6.0)^36^, Glimmer hmm (v3.0.4)^37^ and GeneMark-ET^38^ were used to predict the repeat-masked genome structure. For protein-based homology prediction, the protein sequences of homologous species including *Daphnia pulex* (GCA_021134715.1), *Procambarus virginalis* (GCA_020271785.1), *Fenneropenaeus chinensis* (GCA_019202785.2), *Penaeus japonicus* (GCA_017312705.1), *Penaeus monodon* (GCA_015228065.1), *Litopenaeus vannamei* (GCA_003789085.1), *Portunus trituberculatus* (GCA_017591435.1) and *M. nipponense* (GCA_015104395.1) were downloaded from the NCBI database and aligned against the *E. carinicauda* genome using GeMoMa (v1.7.1)^39^ to perform homology prediction. Furthermore, the RNA-seq data from different tissues and embryonic development stages (PRJNA594425, PRJNA746617, PRJNA756619, PRJNA881755, and PRJNA881756) were mapped to the genome by HISAT2 (v2.1.0)^40^. The full-length transcripts (PRJNA594425) from our previous study^41^ were assembled using Cufflinks (v2.1.1)^42^, then the open reading frame was predicted using PASA (v20140417)^43^. The EVidenceModeler^44^ was employed to consolidate the results from these three methods, enabling the merging and integration of gene predictions. Finally, 44,288 high-quality protein-coding genes were predicted. These predicted genes displayed an average gene length of 28,448 bp, an average coding length of 1,424 bp and 6.09 coding exons per gene.

These genes were functionally annotated using BLAST against NR, SwissProt, eggNOG, InterPro, GO and KEGG^45^. The protein-coding gene functional annotation results were merged using the aforementioned methods. Finally, 70.53% of the total predicted genes were successfully assigned with at least one functional annotation (Table 6).

**Table 6:** Statistical results of gene function annotation.

Database	Number	Percent
Total	44,288	100%
Annotated	31,238	70.53%
NR	31,193	70.43%
SwissProt	16,550	37.37%
KEGG	11,490	25.94%
GO	2,078	4.69%
eggNOG	25,409	57.37%
Unannotated	13,050	29.47%

## Data Records

All sequencing data have been uploaded to the NCBI SRA database. The Illumina sequencing data for genomic survey has been deposited in the NCBI Sequence Read Archive with accession number SRR27880589^46^ under BioProject accession number PRJNA1070324.

The genomic PacBio sequencing data has been deposited in the NCBI Sequence Read Archive with accession number SRR27756800^47^, SRR27756801^48^, SRR27862044^49^ and SRR27862045^50^ under BioProject accession number PRJNA1070324.

The Hi-C sequencing data has been deposited in the NCBI Sequence Read Archive with accession number SRR27880535^51^, SRR27880536^52^, SRR27880537^53^, SRR27880538^54^, SRR27880539^55^ and SRR27880540^56^ under BioProject accession number PRJNA1073006.

The final chromosome-level assembled genome file has been uploaded to the GenBank database under the accession JAZBEV000000000^57^.

## Technical Validation

To evaluate the integrity and accuracy of the genome assembly, the completeness of the final genome assembly was assessed using BUSCO (v5.2.2) and the arthropoda_odb10 database^25^. It was shown that 92.89% of the 1013 single-copy genes were highly conserved orthologs (88.75% complete, 4.15% fragmented, and 7.11% missing). By aligning the Illumina sequencing reads (PRJNA471201)^3^ to the genome using BWA (v0.7.15)^24^, the read-mapping rate was 99.00%. This indicates a high mapping efficiency. Thus, the above results indicated that we obtained a high-quality genome of the *E. carinicauda*.

## Data Availability

No specific code was used in this study. The data analyses used standard bioinformatic tools specified in the methods.
